# Factors related to T1 slope: spinopelvic balance and thoracic compensation

**DOI:** 10.1186/s12893-023-02053-z

**Published:** 2023-05-29

**Authors:** Chengxin Liu, Yongjin Li, Xiangyu Li, Bin Shi, Shibao Lu

**Affiliations:** 1 Department of Orthopedics, Xuanwu Hospital, Capital Medical University, Beijing, China; 2 National Clinical Research Center for Geriatric Diseases, Beijing, China

**Keywords:** Pelvic compensation, Spinopelvic balance, Spine global balance, T1 slope, Thoracic compensation

## Abstract

**Objective:**

To identify factors associated with T1 slope (T1S).

**Methods:**

A total of 215 patients over 18 years old who underwent whole-spine X-rays to evaluate lower back pain were enrolled in this study. T1S, pelvic tilt (PT), sacral slope (SS), pelvic incidence (PI), thoracic kyphosis (TK), lumbar lordosis (LL), cervical lordosis (CL), thoracolumbar kyphosis (TLK), and sagittal vertical axis (SVA) were measured. Patients were divided into balance, compensatory balance, thoracic compensation, and thoracic decompensation groups.

**Results:**

TK (p < 0.001), SVA (p < 0.001), and CL (p = 0.020) were significantly related to high T1S. The balance group had the smallest PT, largest SS and largest LL of the four groups (p < 0.001). The thoracic compensation group had the smallest TK of all groups (p < 0.001). There was no significant difference in T1S between the balance and thoracic compensation groups (p = 0.099). The thoracic decompensation group had a larger T1S than the balance group (p = 0.023).

**Conclusions:**

Caudal spine segments had a sequential effect on cranial spine segments. T1S reflected the compensation ability of the spine. The absence of balance tended to increase the T1S. Pelvic posterior rotation and thoracic compensation were two crucial factors protecting against increased T1S in patients with ASD.

## Introduction

Over the last 20 years, sagittal spine alignment had been studied extensively from the thoracolumbar to the cervical spine [1]. T1 slope (T1S) had received substantial attention because it is the junction of the cervical and thoracolumbar spine. In 2010, [2]Knott et al. proposed that the T1 slope helped evaluate sagittal balance. Subsequently, the T1S was studied in terms of cervical sagittal alignment. Pelvic parameters affect the entire underlying sagittal spinal profile; [3]Lee et al. introduced the notion that thoracic inlet alignment parameters, including T1S, influenced the sagittal balance of the cranium and cervical spine. Ames et al. [4] suggested that mismatch of T1S and cervical lordosis (CL) were significant for cervical deformity. Studies reoprted that extremely high T1S should be considered thoracic deformity or thoracolumbar deformity and suggested correction of the thoracic spine to reduce T1S [1, 5]. Some studies demonstrated that patients with a high T1S had more kyphotic cervical alignment after cervical surgery [6–9]. These findings suggest that T1S is an essential parameter in spine sagittal balance evaluation, surgery planning, and outcome prediction.

Which factors are related to the T1S? There is no standard answer for this question. Inoue et al. [10] reported that the T1S increased with age. Lee et al. [11] found that T1S was influenced by thoracic inlet angle and thoracic kyphosis (TK). Pesenti et al. [12] reviewed adolescent idiopathic scoliosis patients and found that a higher T1S was associated with worse global alignment. However, age, spine global alignment, and thoracic alignment were affected by one another. Deeper insight is needed to clarify the factors related to the T1S.

Therefore, this study aimed to analyze the relationship between spine sagittal parameters and T1S and attempt to identify the factors related to T1S.

## Method

### Patient selection

The institutional review board approved this study of the authors’ affiliated institution. We retrospectively reviewed 215 patients over 18 years old who underwent whole-spine X-rays to evaluate lower back pain between 2019 and 2020 and all patients were followed up for at least three months. Exclusion criteria were congenital spine deformity, neck pain, history of spine surgery, malignancy, or neurological disorders. Demographics including age and sex were recorded.

### Spine radiographic parameters

**Fig. 1:**
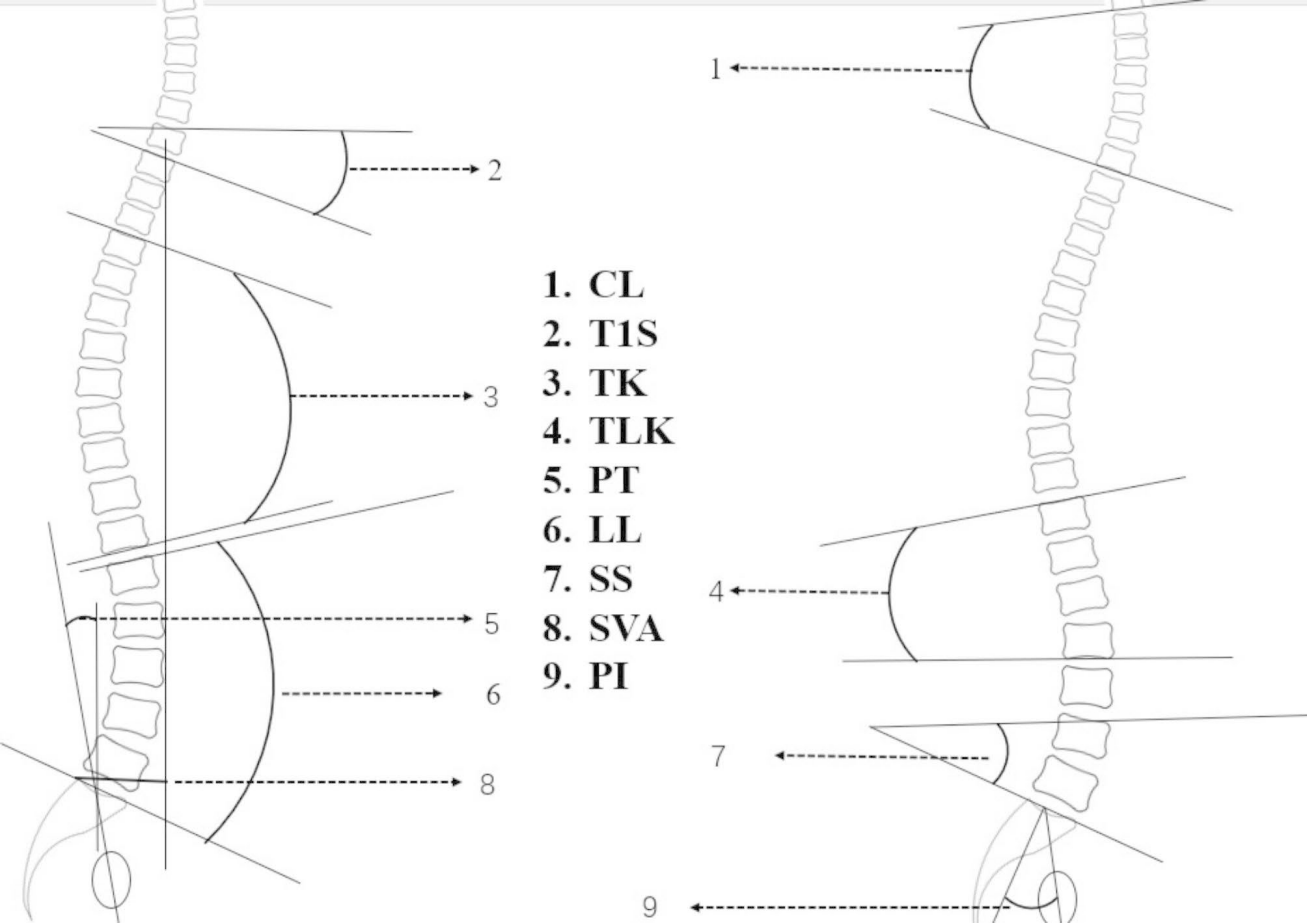
Method to measure spine sagittal alignment PT: pelvic tilt; SS: sacral slope; PI: pelvic incidence; TK: thoracic kyphosis; LL: lumbar lordosis; CL: cervical lordosis; TLK: thoracolumbar kyphosis; SVA: sagittal vertical axis; T1S:T1 slope

Whole-spine standing lateral radiographs were obtained in a standardized upright position. Spine sagittal alignment measurements were defined as follows with neutral standing lateral x-ray images (Fig. 1).

T1S was defined as the angle between a horizontal plane and a line parallel to the superior T1 endplate. Pelvic tilt (PT) was defined as the angle between the line connecting the midpoint of the S1-endplate to the axis of the femoral heads and the vertical plane. Sacral slope (SS) was defined as the angle between the horizontal and sacral endplates. Pelvic incidence (PI) was defined as the angle perpendicular to the sacral endplate at its midpoint and the line connecting this point to axes of the femoral heads. TK was measured from the upper endplate of T4 to the lower endplate of T12. Lumbar lordosis (LL) was defined as the angle between S1-endplate and L1 upper endplate. Cervical lordosis (CL) was measured between the C2 lower endplate and the C7 lower endplate. Thoracolumbar kyphosis (TLK) was measured by the Cobb angle between the upper endplate of T10 and the lower endplate of L2. The C7 sagittal vertical axis (SVA) was defined as the horizontal distance from the superior posterior end of the upper sacral endplate to the C7 plumbline. We defined lordosis as a positive value and kyphosis as a negative value.

### Group classification

**Fig. 2:**
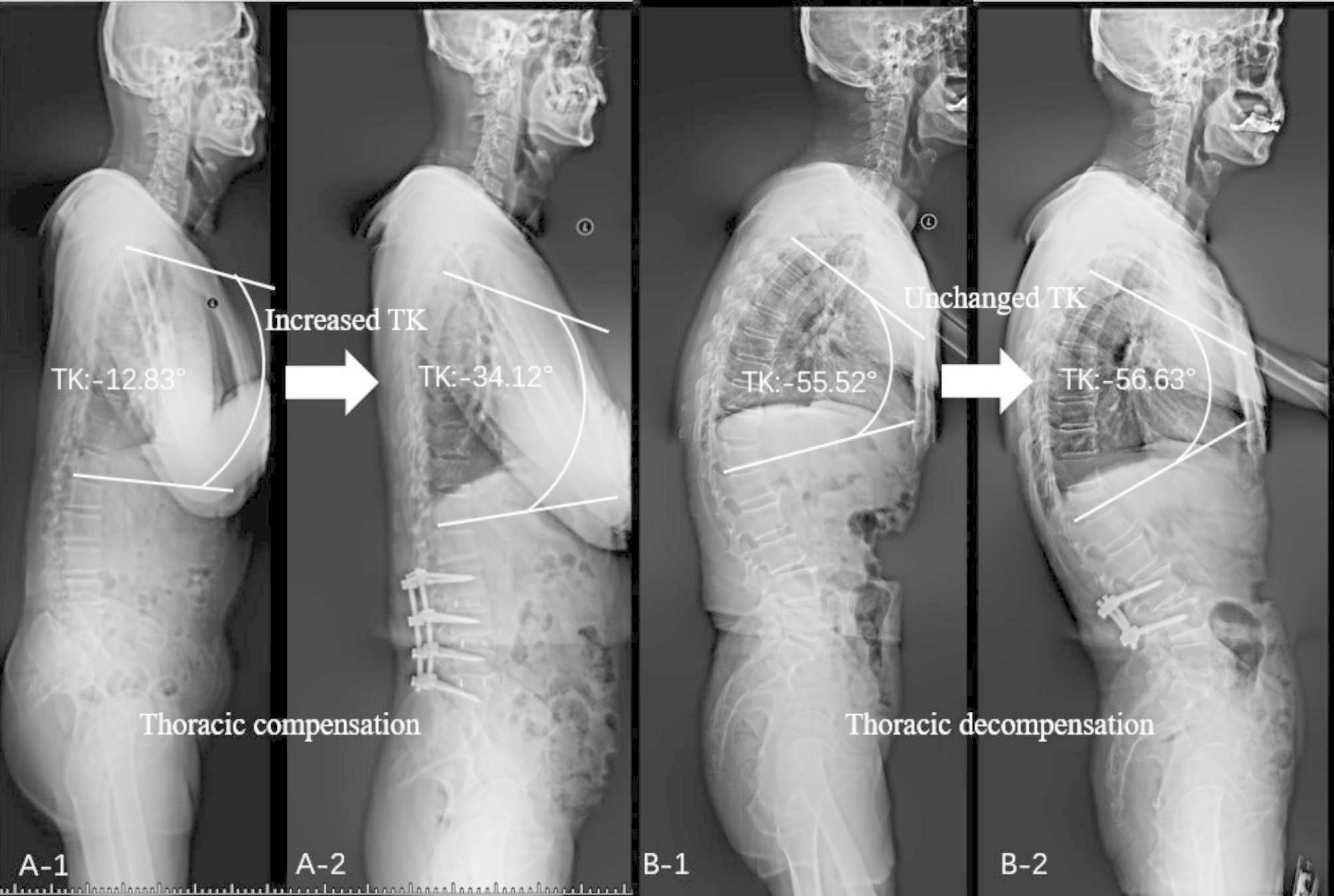
Examples of thoracic compensation (**A**-1,2) and thoracic decompensation (**B**-1,2) groups TK: thoracic kyphosis

**Fig. 3:**
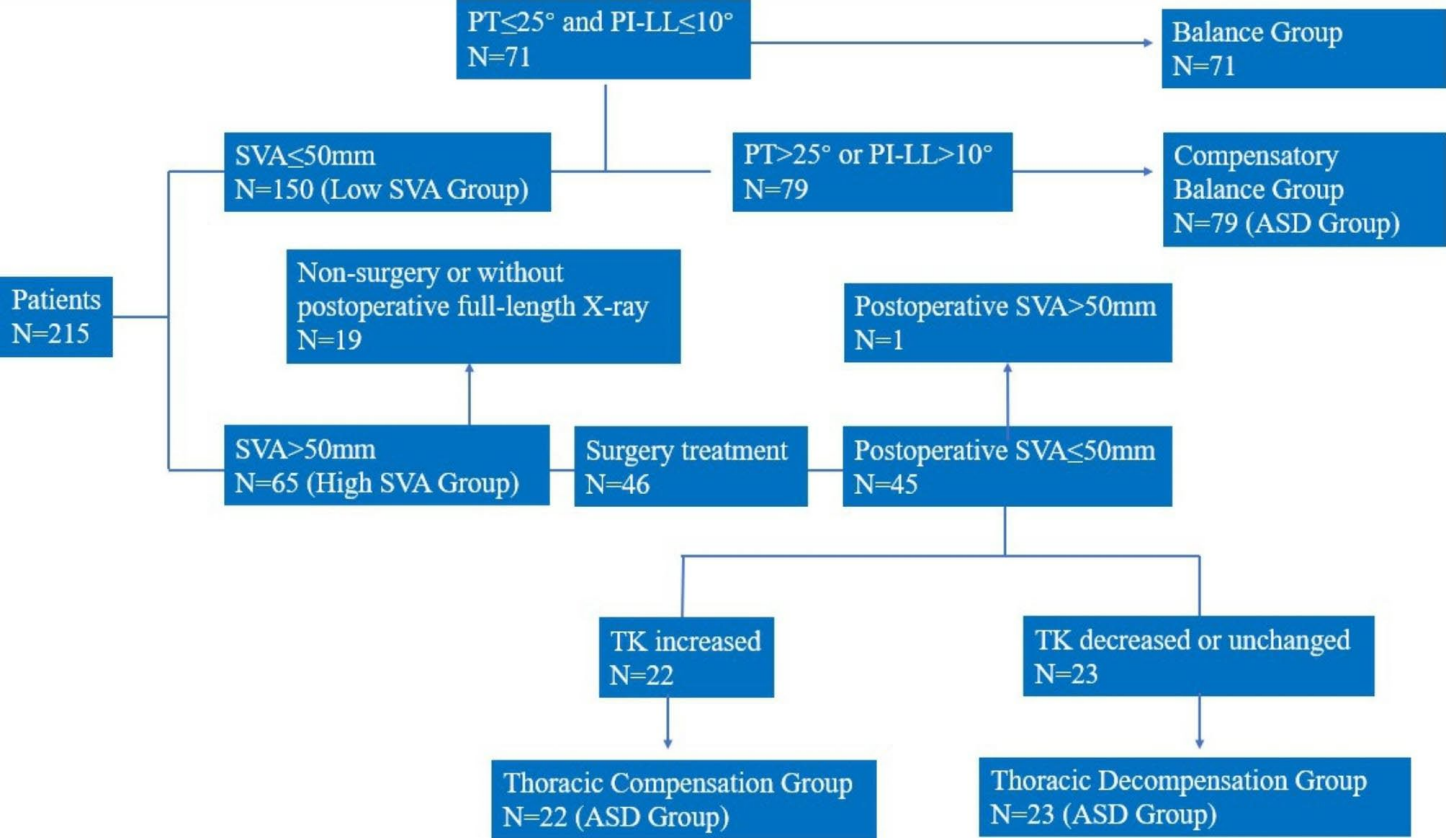
The flowchart of group classification PT: pelvic tilt; PI: pelvic incidence; TK: thoracic kyphosis; LL: lumbar lordosis; SVA: sagittal vertical axis; ASD: adult spinal deformity

According to the T1S, patients were divided into a low T1S group (T1S ≤ 25°) and a high T1S group (T1S > 25°). According to the SVA value, patients were divided into a low SVA group (SVA ≤ 50 mm) and a high SVA group (SVA > 50 mm). In the low SVA group, we evaluated PT and PI-LL. Patients with PT > 25° or PI-LL > 10° were separated into a compensatory balance group and the other patients were separated into a balance group. In the high SVA group, we evaluated postoperative SVA and postoperative TK of patients who underwent lumbar fusion surgery (cranial fusion level below L1) and underwent postoperative full-length X-rays. Patients with postoperative SVA > 50 mm were considered unsatisfactory correction and were excluded. Patients with increased TK (postoperative TK – preoperative TK < -5°) were separated into a thoracic compensation group. Patients with decreased or unchanged TK (postoperative TK – preoperative TK ≥ -5°) were separated into a thoracic decompensation group (Fig. 2). Patients in the compensatory balance and high SVA groups were included in the adult spinal deformity (ASD) group. The flowchart of group classification is displayed in Fig. 3.

### Data analysis

All collected data were analyzed using IBM SPSS Statistics, version 22.0 (IBM Corp., Armonk, NY, USA). Statistical analysis was performed using the t-test, Mann–Whitney U test, Kruskal–Wallis H test, Pearson correlation analysis, one-way analysis of variance, the Tamhane T2 post hoc test, or the Fisher least significant difference post hoc significance test. We performed one-to-one propensity score matching using logistic regression with match tolerance of 0.02 based on covariates include age, sex, and PI value, to adjust for the differences between the groups. The results are presented as the mean value ± standard deviation. A probability (P) value of ≤ 0.05 was considered statistically significant.

## Results

### T1 slope analysis

In this retrospective study, we initially analyzed 215 consecutive patients, including 144 females and 71 males. The mean age was 65.67 ± 11.28 years. We divided 114 patients into the low T1S group. We divided 101 patients into the high T1S group. The demographic data and radiological parameters of the low T1S and high T1S groups are shown in Table 1. Age, TLK, TK, CL, and SVA showed significant differences between the Low T1S and the High T1S group (p < 0.001, p = 0.017, p < 0.001, p < 0.001, and p < 0.001, respectively). The Low T1S group was younger, had a smaller TLK, a smaller TK, a smaller CL, and a smaller SVA. There were no significant differences in sex, PI, PT, SS, or LL between the groups (Table 1).

**Table 1:** Comparison between low T1S and high T1S groups

Parameters	Total (n = 215)	Low T1S (n = 114)	High T1S (n = 101)	P (Low T1S vs. High T1S)
**Age (years)**	65.67 ± 11.28	62.89 ± 10.40	68.82 ± 11.46	< 0.001
**Sex (M/F)**	71/144	33/81	38/63	0.178
**T1S (°)**	26.26 ± 7.87	20.55 ± 3.41	30.34 ± 4.30	< 0.001
**PI (°)**	49.65 ± 9.21	49.93 ± 9.09	49.34 ± 9.38	0.640
**PT (°)**	18.34 ± 9.11	17.86 ± 8.64	18.89 ± 9.63	0.412
**SS (°)**	31.31 ± 9.35	32.07 ± 8.94	30.45 ± 9.77	0.207
**LL (°)**	28.23 ± 16.38	26.35 ± 15.04	30.36 ± 17.60	0.073
**TLK (°)**	-11.70 ± 11.64	-9.93 ± 11.52	-13.70 ± 11.51	0.017
**TK (°)**	-34.66 ± 12.89	-30.18 ± 10.74	-39.71 ± 13.29	< 0.001
**CL (°)**	17.19 ± 12.82	12.86 ± 11.22	22.08 ± 12.79	< 0.001
**SVA (mm)**	28.79 ± 43.89	13.34 ± 37.35	46.23 ± 44.36	< 0.001

T1S: T1 slope; PT: pelvic tilt; SS: sacral slope; PI: pelvic incidence; TK: thoracic kyphosis; LL: lumbar lordosis; CL: cervical lordosis; TLK: thoracolumbar kyphosis; SVA: sagittal vertical axis

We used the Pearson correlation coefficients to analyze the correlations between parameters and T1S (Table 2). T1S had significant correlations with age, TLK, TK, CL, and SVA (Pearson correlation coefficients of 0.261, -0.145, -0.420, 0.487, and 0.394, respectively).

**Table 2:** Pearson coefficients and p-values between parameters and T1 slope

Parameters	Pearson coefficients	P value
**Age**	0.261	< 0.001
**PI**	0.010	0.885
**PT**	0.088	0.196
**SS**	-0.076	0.264
**LL**	0.039	0.566
**TLK**	-0.145	0.034
**TK**	-0.420	< 0.001
**CL**	0.487	< 0.001
**SVA**	0.394	< 0.001

PT: pelvic tilt; SS: sacral slope; PI: pelvic incidence; TK: thoracic kyphosis; LL: lumbar lordosis; CL: cervical lordosis; TLK: thoracolumbar kyphosis; SVA: sagittal vertical axis

To identify the factors associated with high T1S, stepwise regression analysis was performed using the dichotomous variable logistic regression model. We found that TK (OR = 0.915, p < 0.001), SVA (OR = 1.267, p < 0.001), and CL (OR = 1.038, p = 0.020) were significantly related to high T1S (Table 3).

**Table 3:** Stepwise logistic regression for high T1 slope

parameters	Coefficient of regression	Standard error	Wald x^2^	P value	OR	95%CI
**TK**	-0.088	0.017	26.897	< 0.001	0.915	0.885–0.947
**SVA**	0.236	0.049	23.199	< 0.001	1.267	1.150–1.394
**CL**	0.037	0.016	5.449	0.020	1.038	1.006–1.071

TK: thoracic kyphosis; CL: cervical lordosis; SVA: sagittal vertical axis

### Group classification

Of the 215 patients, 150 had an SVA ≤ 50 mm, including 71 patients in the balance group and 79 patients in the compensatory balance group. Sixty-five patients had an SVA > 50 mm, of whom 21 were excluded due to non-surgical treatment or unsatisfactory correction. We analyzed postoperative parameters in 45 patients; 22 were separated into the thoracic compensation group, and 23 were separated into the thoracic decompensation group (Fig. 3).

### Comparison among four groups

We compared demographic data and radiological parameters among the Balance, compensatory balance group, thoracic compensation, and thoracic decompensation group (Table 4). There were no significant differences in sex, PI and TLK among the four groups. The thoracic decompensation group was older than the balance and compensatory balance groups, and the thoracic compensation group was older than the compensatory balance group (p < 0.001). The thoracic decompensation group had a more significant T1S than the balance and compensatory balance groups (p < 0.001). The balance group had a smaller PT than the compensatory balance, thoracic compensation, and thoracic decompensation groups (p < 0.001). The balance group had the largest SS in all groups (p = 0.001). The balance group had the largest LL, and the thoracic compensation group had the smallest TK of all groups (p < 0.001). The thoracic decompensation group had a larger CL than the balance and compensatory balance groups (p = 0.001).

**Table 4:** Comparisons among balance, compensatory balance, thoracic compensation, and thoracic decompensation groups

Parameters	Balance (n = 71)	Compensatory balance (n = 79)	Thoracic compensation (n = 22)	Thoracic decompensation (n = 23)	P value
**Age (years)**	63.06 ± 10.57*	62.82 ± 11.57!@	68.09 ± 9.23!	74.91 ± 7.94*@	< 0.001
**Sex (M/F)**	24/47	23/56	8/14	10/13	0.618
**T1S (°)**	24.23 ± 5.38*	23.29 ± 5.61!@	26.34 ± 7.06!	29.50 ± 6.25*@	< 0.001
**PI (°)**	47.25 ± 8.29	51.74 ± 8.60	49.78 ± 10.12	48.74 ± 9.32	0.062
**PT (°)**	11.91 ± 5.35*!@	20.97 ± 7.21*	23.56 ± 11.13!	20.57 ± 10.66@	< 0001
**SS (°)**	34.34 ± 8.92*!@	30.77 ± 26.22*#	26.22 ± 10.42!#	28.17 ± 9.81@	0.001
**LL (°)**	44.08 ± 9.03*!@	32.43 ± 10.79*#	21.75 ± 12.40!#$	31.28 ± 12.89@$	< 0001
**TLK (°)**	-11.62 ± 12.08	-11.03 ± 10.68	-11.73 ± 11.75	-18.01 ± 13.62	0.086
**TK (°)**	-37.86 ± 10.18*	-33.50 ± 12.55!	-23.29 ± 11.90*!#	-37.70 ± 15.25#	< 0001
**CL (°)**	15.42 ± 12.64*	13.27 ± 10.25!@	19.70 ± 12.60!	23.27 ± 10.74*@	0.001
**SVA (cm)**	-0.06 ± 2.35*!@	1.07 ± 2.59*#	8.23 ± 2.86!#	8.05 ± 2.24@	< 0.001

*, @,!, and # indicated p < 0.05 between the two subgroups

T1S: T1 slope; PT: pelvic tilt; SS: sacral slope; PI: pelvic incidence; TK: thoracic kyphosis; LL: lumbar lordosis; CL: cervical lordosis; TLK: thoracolumbar kyphosis; SVA: sagittal vertical axis

### Comparison between thoracic compensation and balance groups

Twenty patients in balance group were matched with twenty patients in thoracic compensation group using propensity score matching with a match tolerance of 0.02 based on age, sex, and PI (Table 5). The thoracic compensation group had a larger PT, a smaller SS, a smaller LL, a smaller TK, and a larger SVA than the balance group (p < 0.001, p = 0.003, p < 0.001, p < 0.001, p < 0.001, respectively). However, there was no significant difference in T1S between the balance and thoracic compensation groups (p = 0.099).

**Table 5:** Comparison between the thoracic compensation and balance groups

Parameters	Balance (n = 20)	Thoracic compensation (n = 20)	P
**Age (years)**	68.30 ± 10.18	67.85 ± 9.55	0.886
**Sex (M/F)**	8/12	8/12	1
**T1S (°)**	24.41 ± 3.84	27.31 ± 6.63	0.099
**PI (°)**	47.79 ± 8.61	48.41 ± 9.53	0.830
**PT (°)**	10.77 ± 6.55	21.51 ± 9.38	< 0.001
**SS (°)**	37.02 ± 9.67	26.91 ± 10.66	0.003
**LL (°)**	50.07 ± 7.29	22.94 ± 12.29	< 0.001
**TLK (°)**	-16.10 ± 11.28	-11.98 ± 12.32	0.276
**TK (°)**	-40.66 ± 8.04	-23.86 ± 12.26	< 0.001
**CL (°)**	17.07 ± 11.52	20.48 ± 12.98	0.385
**SVA (cm)**	-0.18 ± 2.43	7.94 ± 2.71	< 0.001

T1S: T1 slope; PT: pelvic tilt; SS: sacral slope; PI: pelvic incidence; TK: thoracic kyphosis; LL: lumbar lordosis; CL: cervical lordosis; TLK: thoracolumbar kyphosis; SVA: sagittal vertical axis

### Comparison between the thoracic decompensation and balance groups

Fourteen patients in balance group were matched with fourteen patients in thoracic decompensation group using propensity score matching with a match tolerance of 0.02 based on age, sex, and PI (Table 6). The thoracic decompensation group had a larger T1S, a smaller LL, a larger CL, and a larger SVA than the balance group (p = 0.023, p = 0.001, p = 0.047, p < 0.001, respectively). There were no significant differences in PT, SS, TLK, or TK between the balance and thoracic decompensation groups (p = 0.062, p = 0.069, p = 0.475, and p = 0.515, respectively).

**Table 6:** Comparison between the thoracic decompensation and balance groups

Parameters	Balance (n = 14)	Thoracic decompensation (n = 14)	P
**Age (years)**	72.29 ± 5.59	72.71 ± 8.20	0.873
**Sex (M/F)**	7/7	5/9	0.541
**T1S (°)**	24.10 ± 5.53	29.04 ± 5.29	0.023
**PI (°)**	46.56 ± 8.72	46.00 ± 8.81	0.866
**PT (°)**	12.18 ± 5.21	17.34 ± 8.45	0.062
**SS (°)**	34.39 ± 7.26	28.66 ± 8.68	0.069
**LL (°)**	46.44 ± 7.10	31.41 ± 13.00	0.001
**TLK (°)**	-16.89 ± 13.96	-20.74 ± 14.18	0.475
**TK (°)**	-43.41 ± 9.26	-40.00 ± 16.94	0.515
**CL (°)**	15.59 ± 9.35	24.38 ± 12.69	0.047
**SVA (cm)**	0.71 ± 2.64	7.38 ± 1.94	< 0.001

T1S: T1 slope; PT: pelvic tilt; SS: sacral slope; PI: pelvic incidence; TK: thoracic kyphosis; LL: lumbar lordosis; CL: cervical lordosis; TLK: thoracolumbar kyphosis; SVA: sagittal vertical axis

## Discussion

Spine sagittal alignment has received focus over the past two decades [1]. The T1 vertebral body, fixed by the two sides of the rib, is the junction of a transitional region between the mobile, lordotic cervical spine and the rigid, kyphotic thoracic spine, all of which cause potential instabilities. T1S, as a parameter reflecting sagittal morphology of the T1 vertebral body, is associated with whole sagittal balance. The T1S larger than 25° or less than 13° indicated that the spine was unbalanced [2]. Studies found that the T1S had a linear relationship with CL [13–15]. These findings suggested that the T1S is an essential parameter in assessing cervical and global spinal sagittal balance [12, 16–18]. The value of the T1S is fundamental for surgical planning and outcome prediction [5, 8, 9, 19–23]. According to previous studies, age, spine global alignment, and thoracic alignment were associated with T1S [2, 11, 12, 24]. We initially analyzed 215 consecutive patients in the present study and found that older age, larger TLK, larger TK, larger CL, and larger SVA were associated with a high T1S. Pearson correlation analysis showed that the T1S had significant correlations with age, TLK, TK, CL, and SVA. Dichotomous variable logistic regression showed that TK, SVA, and CL were significantly related to the high T1S. These findings suggested that the T1S was influenced by lower adjacent segments and global spine alignment. CL compensated for the T1S to maintain horizontal gaze. These results were consistent with a previous study demonstrating that caudal spine segments had a sequential effect on cranial spine segments [25].

Some studies found that the T1S increased with age [10, 26]. However, there are also spine sagittal parameters other than age that have relationships with the T1S. Several studies reported the relationships between T1S and other global spine parameters. Lee et al. [1, 3] found a significant relationship between T1S and TK. A high T1S was related to a large CL to maintain the sagittal balance of the cervical spine [27]. We believe that age-related degenerative changes of the spine explained T1S change. Aging spines lose the characteristic shape of the vertebral body disks resulting in decreased lumbar lordosis or thoracolumbar kyphosis. Compensatory mechanisms for degenerative changes might influence the T1S.

**Fig. 4:**
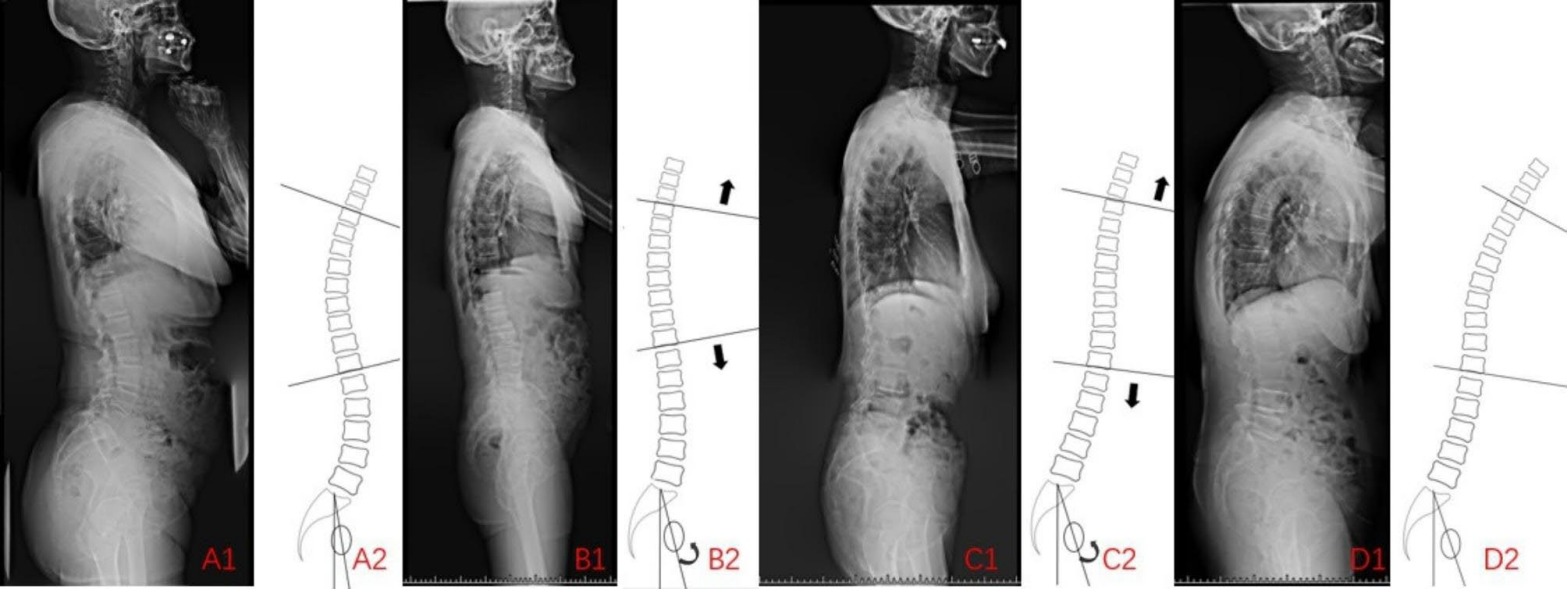
Examples and compensation mechanism diagrams of the balance (**A** 1,2), compensatory balance (**B** 1,2), thoracic compensation (**C** 1,2), and thoracic decompensation (**D** 1,2) groups

We used a four-group classification to analyze the influence of ASD and thoracic compensation on the T1S. Compared with the Balance group, compensatory balance group and high SVA groups had lower LL and greater PT, suggesting that posterior pelvic rotation occurred in three ASD groups to compensate for lumbar deformity. Because of reasonable compensation in the compensatory balance group, the T1S was slightly reduced and similar to the balance group. In the high SVA group, the thoracic compensation group had decreased TK, restricting T1S enlargement. There was no significant difference in T1S between the balance and thoracic compensation groups. According to propensity score matching, compared with the balance group, the thoracic compensation group had posterior pelvic rotation and decreased TK. The increased T1S in the thoracic compensation group was not significantly different from the balance group. However, the thoracic decompensation group had no extra compensation ability to reduce the T1S. According to propensity score matching, compared with the balance group, the thoracic decompensation group showed limited pelvic posterior rotation, limited TK decrease, but increased T1S. These findings suggested a significant difference in T1S between the thoracic decompensation and balance groups. In our opinion, posterior pelvic rotation and thoracic compensation are two crucial factors protecting against T1S increases (Fig. 4).

Extension of the thoracic spine and pelvic retroversion are essential mechanisms to prevent the center of gravity from moving forward. Garbossa et al. [28]reported three types of spinal alignment, and active compensation mechanisms in the hidden imbalance state could maintain the balance of the global spine. However, an imbalanced spine state had anterior gravity lines despite active compensation mechanisms. In the present study, the compensatory balance group maintained the global spine and T1S balance well because of reasonable compensation. Extension of the thoracic spine in the thoracic compensation group prevented the T1S from over enlargement despite the balance of the global spine being destroyed. The thoracic decompensation group could not prevent the increase in T1S because of lack of compensation. In other words, the T1S reflected the compensation ability of the spine. Patients with high T1S might lack compensation ability for spine degeneration. Studies reported that high T1S induced postoperative kyphotic change after cervical laminoplasty [7–9, 19–21, 29]. These studies may help to explain that the poor outcome of high T1S patients after surgery may be related to lack of compensation.

This study has several limitations. First, a large sample cohort with long-term follow-up is more suitable to address this question. Second, lower limb parameters were not evaluated because of the range of whole-spine radiography. Finally, more deep studies are needed to validate our results further. Nevertheless, we believe that our results help understand the value of the T1 slope.

## Conclusion

Caudal spine segments had a sequential effect on cranial spine segments. TK, SVA, and CL were highly related to the T1S. T1S reflected the compensation ability of the spine. Unbalanced spines tended to increase T1S. Pelvic posterior rotation and thoracic compensation were two crucial factors protecting against increased T1S in patients with ASD.

## Data Availability

All data generated or analyzed during this study are included in this published article and its supplementary information files.

## Abbreviations

T1S: T1 slope
CL: Cervical lordosis
PT: Pelvic tilt
SS: Sacral slope
PI: Pelvic incidence
TK: Thoracic kyphosis
LL: Lumbar lordosis
TLK: Thoracolumbar kyphosis
SVA: C7 sagittal vertical axis
ASD: Adult spinal deformity
