# Correction: Factors related to T1 slope: spinopelvic balance and thoracic compensation

**DOI:** 10.1186/s12893-023-02074-8

**Published:** 2023-06-27

**Authors:** Chengxin Liu, Yongjin Li, Xiangyu Li, Bin Shi, Shibao Lu

**Affiliations:** 1 Department of Orthopedics, Xuanwu Hospital, Capital Medical University, Beijing, China; 2 National Clinical Research Center for Geriatric Diseases, Beijing, China

**Correction: BMC Surg 23, 145 (2023)**

**https://doi.org/10.1186/s12893-023-02053-z**

Following publication of the original article [1], In this article Chengxin Liu, Yongjin Li and Xiangyu Li should have been denoted as an equally contributing authors.

The original article has been corrected.
